# Serological profile among patients with malaria in secondary community hospitals in India - a multicenter cross sectional study

**DOI:** 10.1186/1475-2875-13-S1-P63

**Published:** 2014-09-22

**Authors:** Kristine Mørch, Rosario Vivek, Depeeka Xena, Sara Chandy, Anand Manoharan, Christel Haanshuus, Nina Langeland, Bjørn Blomberg, Dilip Mathai

**Affiliations:** 1 Haukeland University Hospital, Bergen, Norway; 2 University of Bergen, Bergen, Norway; 3 Christian Medical College, Vallore, India

## Background

Malaria symptoms are unspecific in acute undifferentiated fever. Seropositivity against various previous infections might be high in exposed populations. Serological results in a cohort of malaria patients in India are presented.

## Materials and methods

Serological tests from patients with malaria in an observational fever study were analyzed. During April 2011-November 2012, 1564 patients aged ≥5 years with fever for 2-14 days were consecutively included at seven secondary community hospitals in Tezpur (Assam), Raxaul (Bihar), Mungeli (Chhattisgarh), Ratnagiri (Maharashtra), Anantapur (Andhra Pradesh), Oddanchatram and Ambur (Tamil Nadu). Scrub typhus IgM ELISA (In Bios, USA), Leptospira IgM ELISA (Alere, Australia), Chikungunya IgM ELISA (NIV, India), Dengue rapid NS1 antigen and IgM Combo test (SD bioline, USA) were performed at the hospital sites. At the monitoring site (Benjamin M Pulimood Laboratories, Christian Medical College, Vellore, India), malaria genus mitochondrial PCR was performed on all samples and 18S species-specific PCR or sequencing performed on genus positive samples with methods as described previously (Haanshuus *et al.*, *Malaria Journal* 2013). At the monitoring site Dengue IgM capture ELISA (MAC-ELISA), Scrub typhus immunofluorescence (IFA) on Scrub ELISA positive samples and Leptospira Microscopic Agglutination Test (MAT) on Leptospira ELISA positive samples were also performed. Convalescence samples for test of rise in titer were not collected due to logistical challenges. Malaria PCR was considered gold standard for the diagnosis of malaria.

## Results

Among malaria cases (N = 268/1564), 15% (173/268) had positive Dengue MAC-ELISA, 14% (37/268) positive Leptospira MAT, 8% (29/233) positive Chikungunya IgM ELISA and 35% (25/71) positive Scrub typhus IFA. Both Chikungunya ELISA and Dengue MAC were positive in 6/268, both Leptospira MAT and Dengue MAC in 4/268, both Scrub typhus IFA and Leptospira MAT in 6/268, both Scrub typhus IFA and Chikungunya ELISA in one and both Scrub typhus IFA, Dengue MAC and Chikungunya ELISA also positive in one patient.

## Conclusions

Serological tests generally considered gold standard for the diagnoses of scrub typhus, leptospirosis, Dengue fever and chikungunya were positive in a high number of malaria patients. Multiple etiologies of fever in addition to malaria to such an extent are not likely, while persistent antibodies after previous infections or cross reactivity more likely explain the findings. Results of a single serological test should be interpreted with caution when treating patients with acute undifferentiated fever in India and malaria should be considered a differential diagnoses also when IgM antibodies against other pathogens are detected.

